# Quantification of *Plasmodium*-host protein interactions on intact, unmodified erythrocytes by back-scattering interferometry

**DOI:** 10.1186/s12936-015-0553-2

**Published:** 2015-02-21

**Authors:** Phoonthawee Saetear, Abigail J Perrin, S Josefin Bartholdson, Madushi Wanaguru, Amanda Kussrow, Darryl J Bornhop, Gavin J Wright

**Affiliations:** Department of Chemistry and Center of Excellence for Innovation in Chemistry, Faculty of Science, Mahidol University, Rama VI Road, Bangkok, 10400 Thailand; Cell Surface Signalling Laboratory and Malaria Programme, Wellcome Trust Sanger Institute, Hinxton, Cambridge, CB10 1SA, UK; Department of Chemistry and the Vanderbilt Institute for Chemical Biology, Vanderbilt University, 4226 Stevenson Center, Nashville, Tennessee 37235 USA

**Keywords:** Backscattering interferometry, Biosensors, Glycophorin A, PfEBA175, Basigin, PfRH5, *Plasmodium falciparum*, Merozoite, Recombinant proteins, Malaria

## Abstract

**Background:**

Invasion of host erythrocytes by *Plasmodium falciparum* is central to the pathogenesis of malaria. Invasion involves recognition events between erythrocyte receptors and ligands on the merozoite, the invasive blood form of the parasite. Identifying and characterizing host-parasite interactions is impeded by the biochemical challenges of working with membrane-embedded glycoprotein receptors. For example, the interaction between *P. falciparum* erythrocyte binding antigen 175 (PfEBA175) and glycophorin A (GYPA) depends on post-translational modifications that are not easily added in recombinant expression systems, and the use of native GYPA is limited by the hydrophobic transmembrane region, making it difficult to biochemically manipulate. It would, therefore, be desirable to perform quantitative binding assays with receptors embedded within the membranes of intact human erythrocytes.

**Methods:**

The extracellular region of GYPA was over-expressed as a soluble protein in HEK293E cells. This protein was characterized, sialylated and evaluated for binding to the PfEBA175 protein. The label-free and free-solution assay, backscattering interferometry (BSI), was used to perform binding assays of two well-characterized *P. falciparum* invasion ligands to intact unmodified human erythrocytes.

**Results:**

Findings indicate that the post-translational modifications present on native GYPA are required for it to bind recombinant PfEBA175 and that these modifications cannot be recapitulated *in vitro* using mammalian overexpression methods. Here, BSI was used to obtain quantitative, high fidelity interaction determinations on intact, unmodified erythrocytes. Using BSI and purified recombinant proteins constituting the entire ectodomains of the *P. falciparum* merozoite ligands PfEBA175 and PfRH5, *K*_D_s of 1.1 μM and 50 nM were measured for the PfRH5-BSG and PfEBA175-GYPA interactions, respectively, in good agreement with previous biophysical measurements of these interactions.

**Conclusions:**

These results demonstrate that BSI can be used to detect and quantify the interactions of two merozoite invasion ligands with their receptors on intact human erythrocytes. BSI assays were performed on unlabelled, free-solution proteins in their native environment, requiring only nanomoles of recombinant protein. This study suggests that BSI can be used to investigate host-parasite protein interactions without the limitations of other assay platforms, and therefore represents a valuable new method to investigate the molecular mechanisms involved in erythrocyte invasion by *P. falciparum*.

## Background

Malaria is an infectious disease caused by several species of protozoan parasites belonging to the *Plasmodium* genus, although just one species, *Plasmodium falciparum*, is responsible for the vast majority of human mortality and morbidity. About half of the world’s population live in regions that are described as being at risk of malaria, with approximately one million deaths attributed to this disease annually [1]. *Plasmodium* parasites are transmitted to the human host through the bite of an infected mosquito and, after a largely asymptomatic liver stage, are released as merozoites, initiating the blood stage of infection. It is during this stage that the symptoms of malaria are exhibited. Once within the blood, *P. falciparum* merozoites recognize and invade host erythrocytes where they multiply and, after rupturing the host cell, are released to begin another cycle of invasion. Since erythrocyte invasion by the *P. falciparum* merozoite is an essential step in the parasite lifecycle, considerable research has been aimed at understanding the molecular mechanisms for the invasion process [2] with the ultimate aim of developing anti-malarial drugs and vaccines [2-4].

It is known that the invasion of erythrocytes by the merozoite involves a series of extracellular molecular recognition events between host receptors and parasite ligands. For *P. falciparum*, several host-parasite receptor-ligand interactions have been described which include the interactions between glycophorin A (GYPA) and erythrocyte binding antigen (EBA)175 [5-8] and those between basigin (BSG) and reticulocyte binding-like homologue (RH)5 [9]. Despite this progress, the molecular mechanisms of invasion are not completely understood and further progress will be facilitated by the application of new methods, especially since the strategies used to date have largely relied on erythrocytes from patients with rare blood groups [6,10-12]. Working with erythrocyte cell surface proteins, however, is technically challenging due to the genetic intractability of the anucleate erythrocyte and difficulties in solubilizing amphipathic membrane proteins in their native conformation. Furthermore, extracellular protein interactions can be highly transient – often having half-lives of just fractions of a second [13]. To address these challenges, a method called AVEXIS (for AVidity-based EXtracellular Interaction Screen) was developed which detects even very weak interactions between the entire ectodomains of receptor proteins expressed as soluble recombinant proteins in mammalian cells [14]. This approach, together with other advances in the expression of full-length, active *P. falciparum* recombinant proteins [15,16], allowed the successful identification of two new erythrocyte-merozoite interactions [9,17]. This progress aside, the majority of the proteins located on the surface of the *P. falciparum* merozoite have no known binding partner [2].

The lack of knowledge about these parasite proteins can be largely attributed to technical limitations of existing approaches that modify the interacting species, or require removing the membrane protein from its native environment [18]. Surface plasmon resonance (SPR) and biolayer interferometry (BLI) have exhibited only a limited number of successes with membrane proteins due to the need to immobilize one of the binding partners and their inherent mass sensitivity. Micro-scale thermophoresis (MST) [19] normally requires removing the protein from the native environment, and the AVEXIS technique is not suitable for proteins where the extracellular region cannot be expressed as a soluble recombinant protein - such as those that span the membrane multiple times; for example, transporters or channels. With over 50 different multi-span membrane proteins detectable on human erythrocytes [20], many potential host receptors are therefore excluded from the AVEXIS approach. Also, AVEXIS, SPR, BLI, and MST are not generally suitable to detect interactions with receptors that are formed by two or more proteins within the membrane. Finally, and as demonstrated here, the requirement to express the proteins recombinantly in a heterologous cell line can result in the absence of cell-specific, post-translational modifications that are essential for ligand binding. With this in mind, a technique that can sensitively and quantitatively measure interactions between *Plasmodium* proteins and their receptors on the surface of intact erythrocytes would be particularly useful.

It has been shown recently that BSI is a highly sensitive, label-free and free-solution assay technology that is compatible with a wide array of complex matrices [21]. This unique interferometer is based on illuminating a 100 × 210-μm dimensioned semicircular channel in a microfluidic chip with coherent light from a laser to create a pattern of light that is interrogated in the backscatter configuration [22]. This pattern of light - the interference pattern - has periodic dim and bright spots (fringes) whose positions are related to the refractive index (RI) of the fluid in the channel. When molecules interact, the compound formed has a distinct and different RI than either of, or the sum of, the RI for the reactants. These changes in RI are a consequence of the conformation, hydration and electron density changing when the ligand binds to the receptor, and induce measurable changes in the position of the interference fringe pattern. BSI has been used to quantify binding affinities on systems with *K*_D_ values ranging from a few picomolar (pM) to tens of millimolar (mM). BSI is a universal detection method which uses control and reference samples to enable the quantification of specific binding reactions, even within highly complex matrices, such as vesicles prepared from cell membranes [21].

Here, it is shown for the first time that BSI can be used to detect and quantify low-affinity interactions between recombinant *Plasmodium* proteins and their known receptors natively embedded within the membrane of whole unmodified erythrocytes. These data demonstrate that composition and environment are critical when attempting to define the molecular mechanisms involved in erythrocyte invasion. BSI enables binding studies to be performed on the intact cell, such that the membrane protein receptors are unaltered and can form complexes, as they would *in vivo*. The results presented here indicate that this BSI-based methodology represents a new approach to identify and quantify interactions involved in erythrocyte invasion by *P. falciparum*, particularly those that contain erythrocyte-specific post-translational modifications.

## Methods

### Recombinant protein production and purification

Recombinant human GYPA, PfEBA175 and PfRH5 proteins were produced by transient transfection of HEK293E cells, as described previously [15]. In brief, the entire ectodomains of PfEBA175 and PfRH5 were codon-optimized for expression in human cells, and cloned into expression plasmids that contained both an N-terminal signal sequence peptide and C-terminal rat Cd4 domain 3 + 4 and hexa-His tags [23]. In the case of human GYPA, the endogenous signal peptide was used and expressed as either an enzymatically biotinylated form or as a β-lactamase tagged pentamer [15]. Capture of β-lactamase tagged pentamers was measured by absorbance of a nitrocefin hydrolysis product at 485 nm. Where appropriate, plasmids encoding the human α-2,3-sialyltransferase 1 (α2,3 ST, NP_003024.1), which catalyzes the synthesis of the Neu5Acα2-3Gal sequence on O-linked glycans, either singly or in combination with the rat α-2,6-sialyltransferase 1 (α-2,6 ST, NP_001106815.1), or the human CMP-sialic acid transporter (SA transporter, NP_006407.1) were co-transfected with the GYPA expression construct in a 1:5 ratio. When the SA transporter was co-transfected, the culture medium was additionally supplemented with 20mM sialic acid precursor, N-acetyl-D-mannosamine. Expression plasmids are openly available from Addgene [24]. Proteins were purified from spent cell culture supernatant using HisTrap HP columns (GE Healthcare) on an ÄKTAxpress instrument (GE Healthcare) as described [17]. Purified proteins were dialyzed extensively against PBS to replace the imidazole-containing purification elution buffer.

### Backscattering interferometry instrumentation

A backscattering interferometer was assembled and used essentially as previously described [22]. To summarize, the instrument is comprised of a helium-neon (He-Ne) laser, a microfluidic chip and a charge-coupled device (CCD) camera acting as a linear array detector. The chip contains flow channels with a semicircular cross-section that, upon proper illumination with the laser, create an optical resonance phenomenon. As a result, BSI has a long effective optical path-length through the sample leading to high RI sensitivity. The backscattered interferometric fringe pattern is directed onto the camera, which in concert with a Fourier analysis programme, is used to measure the positional shift in the fringes. This phase value (in radians) is used to define or quantify the changes in RI between a control and a test sample. Test and control samples are sequentially introduced into a microfluidic chip and allowed to reach temperature and pressure equilibrium (~10 sec), at which point the phase value is recorded. The relative RI changes between the test sample and non-binding controls are correlated with the conformation changes resulting from a molecular interaction. By fixing the concentration of the receptor (number of erythrocytes) and sequentially increasing the concentration of the ligand in endpoint equilibrium binding assays, the measured phase changes between the test and control samples can be used to construct binding isotherms, from which equilibrium dissociation constants (*K*_D_s) can be calculated.

### Erythrocyte preparation

A-positive erythrocytes (Valley Biomedical) were prepared to 2% haematocrit in RPMI 1640 medium (Gibco) either with or without 20 mU/mL neuraminidase (Sigma Aldrich). Erythrocytes were then incubated for one hour at 37°C with gentle agitation. Cells were washed twice with PBS, and resuspended to 1% haematocrit in PBS-containing protease inhibitors (Roche).

### Binding assays using BSI

All binding assays were performed at 0.1% haematocrit in 0.01% (w/v) BSA-PBS buffer containing serial dilutions of the *Plasmodium* proteins ligands (PfEBA175 or PfRH5) as appropriate. The BSA in the buffer helped to decrease adsorption of samples to the channel surface. All samples were incubated at room temperature for one hour to allow binding equilibrium to be reached. Upon injection of each sample, the phase of the fringe pattern was recorded and phase changes were calculated by subtracting the phase measurement recorded for a control sample from a matched test sample. The channel was washed with 30 μL BSA-PBS buffer between samples. When required, channels were cleaned more thoroughly using serial injections of 10 μL methanol (50% (v/v)), 30 μL water and finally 30 μL BSA-PBS. Saturation binding curves were generated by plotting the phase change between test and reference against the concentration of *Plasmodium* protein used. Determination of the equilibrium dissociation constant (*K*_D_) was carried out by fitting the saturation-binding isotherm as a square hyperbolic using GraphPad Prism software.

To quantify the binding of PfEBA175 to GYPA on intact erythrocytes, either neuraminidase-treated cells or erythrocytes pre-incubated with an anti-GYPA monoclonal antibody were used as reference samples. In both cases, the responses of these references were subtracted from phase values obtained from untreated erythrocytes to obtain the phase change due to binding. For antibody blocking, erythrocytes were incubated with a final concentration of 100 μg/mL BRIC256 (Abcam) at room temperature for one hour, since this antibody had previously been shown to prevent the binding of PfEBA175 to GYPA [8]. Neuraminidase-treated and antibody-blocked (control samples) and untreated (test) erythrocytes were incubated with a range of concentrations of PfEBA175 up to 1 μM. Similarly, to quantify the binding of PfRH5 to BSG on the erythrocyte surface, erythrocytes were incubated with 10 μg/mL of the MEM-M6/6 anti-BSG monoclonal antibody (Abcam) at room temperature for one hour. This antibody is known to block the interaction between PfRH5 and BSG and can prevent *P. falciparum* parasites from invading erythrocytes [9]. Antibody-blocked (control) and untreated (test) erythrocytes were incubated with a range of concentrations of PfRH5 up to 4.5 μM.

## Results

### Recombinant glycophorin A expressed in HEK293E cells is undersialylated and does not bind PfEBA175

**Figure 1:**
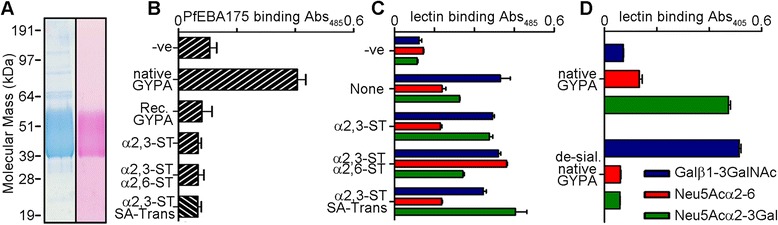
**Recombinant soluble GYPA expressed in HEK293E cells is undersialylated and does not bind PfEBA175. (A)** Denaturing SDS-PAGE gels of recombinant soluble GYPA, visualized using Coomassie G250 (left panel) and a glycoprotein-specific periodic acid-Schiff reagent stain (right panel). **(B)** Recombinant soluble biotinylated GYPA co-expressed without (Rec. GYPA) or with plasmids encoding α-2,3 sialyltransferase 1 (α2,3-ST) singly or in combination with an α-2,6 sialyltransferase 1 (α2,6-ST) or a CMP-sialic acid transporter (SA-Trans) were immobilized on a streptavidin-coated plate and tested for their ability to bind pentameric β-lactamase-tagged PfEBA175. The positive control was biotinylated native GYPA and the negative control (−ve) was biotinylated Cd4d3 + 4 alone. **(C)** Binding of pentameric β-lactamase-tagged recombinant GYPA and its sialylation-enhanced forms by the three lectins that recognize terminal sialic acids (Neu5Ac) or a subterminal Galβ1-3GalNAc disaccharide. Negative control was soluble pentameric β-lactamase-tagged Cd4d3 + 4. The assay was a modified version of AVEXIS with the biotinylated lectins immobilized on a streptavidin-coated plate. **(D)** Lectin binding to native GYPA and its desialylated (neuraminidase-treated) form to show extent of sialylation of native GYPA. Figure 1D is reproduced from Wanaguru *et al*. with minor stylistic changes [8]. Bars represent mean ± standard deviation (SD), *n* = 3.

The entire ectodomain of PfEBA175 was previously expressed as a soluble recombinant protein in HEK293E cells and shown to interact with its receptor, GYPA, on erythrocytes in a manner that is neuraminidase-sensitive [8]. While recombinant PfEBA175 protein also bound native full-length GYPA extracted from erythrocytes, full-length GYPA is of limited use for *in vitro* functional assays because the hydrophobic transmembrane region makes it difficult to biochemically manipulate. Therefore, just the extracellular regions of GYPA were expressed as a soluble recombinant protein in HEK293E cells. Even though this approach resulted in a highly glycosylated protein of the expected mass (Figure 1A) which bound anti-GYPA monoclonal antibodies suggesting it was correctly folded, recombinant GYPA did not bind PfEBA175 (Figure 1B). Since under-sialylation of recombinant proteins overexpressed in HEK293 cells has been previously reported [25], it is likely that GYPA was unable to bind PfEBA175 due to inadequate sialylation. To increase the level of sialylation of the recombinant GYPA, it was co-transfected with plasmids encoding α-2,3-sialyltransferase 1, α-2,6-sialyltransferase 1, or the human CMP-sialic acid transporter. Lectin binding assays did show that the level of sialylation of recombinant GYPA was increased (Figure 1C), yet no treatment was sufficient to attain the same level of sialylation as is found in native GYPA (Figure 1D) and they did not bind to PfEBA175 (Figure 1B).

Taken collectively, these findings demonstrate, at least in the case of the PfEBA175-GYPA interaction, that the environment and the post-translational modifications present on the native protein are critical and required for ligand binding. This result is perhaps not surprising given examples within the literature illustrating the often misleading results that can be obtained when working with protein fragments or in non-native environments. For the PfEBA175-GYPA interaction, the ideal approach would be to employ a method that could quantify PfEBA175-binding on native GYPA presented on whole, unmodified erythrocytes. Since BSI showed efficacy for other membrane protein binding assays, it might be possible to measure interactions using intact erythrocytes using this method.

### Backscattering interferometry can detect and quantify PfEBA175 binding to GYPA on whole, unmodified erythrocytes

**Figure 2:**
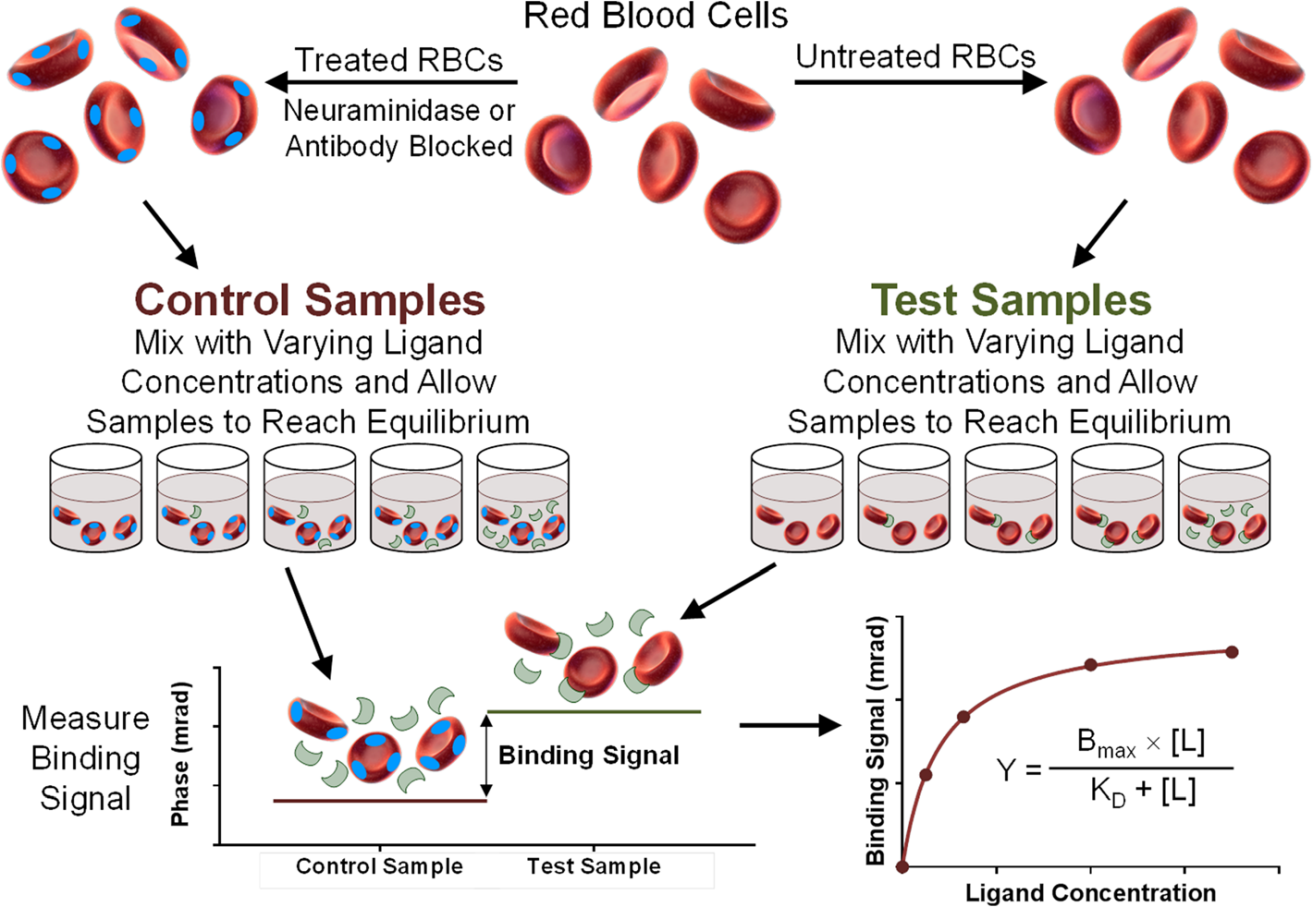
**Using backscattering interferometry to quantify** ***Plasmodium*** **-host protein interactions on intact**, **unmodified erythrocytes.** A schematic illustrating how the BSI method was used to detect and quantify *Plasmodium*-host protein interactions at the surface of intact erythrocytes. Erythrocytes are used either unmodified (test samples), or modified to produce a non-binding reference (control samples) either by blocking specific receptors by pre-incubation with monoclonal antibodies (blue ovals), or, in the case of the PfEBA175-GYPA interaction, treated with neuraminidase. Both the control and test samples are independently incubated with varying concentrations of recombinant soluble *Plasmodium* ligands (green crescents) until binding equilibrium is reached and the binding signal is then quantified by backscattering interferometry. For each ligand concentration, the phase shift response in the control sample is subtracted from that in the test sample and plotted as a function of the ligand concentration. The equilibrium binding constant is calculated by curve fitting using a square hyperbolic function to the binding isotherm.

BSI is a universal, optical, label-free, free-solution method and, as such, to quantify specific binding signals, the response of a non-binding control sample (one that has the same RI composition except for the binding ligand) is used to quantify specific binding reactions within complex matrices. Thus, the fringe shift of the control was subtracted from that of the test sample, which contains both the target and ligand (see Figure 2 for an explanatory schematic).

Since BSI has never been used to quantify interactions with endogenously expressed receptors, because of the high abundance of the GYPA receptor on erythrocytes, and the inability to detect binding of PfEBA175 to any of the recombinant forms of GYPA in the AVEXIS assay, the PfEBA175-GYPA interaction represented a good test system for a first attempt to use BSI to detect binding of recombinant protein to native membrane-protein on intact human erythrocytes. The dependence of this interaction on the presence of sialic acid [5], facilitated treating erythrocytes with neuraminidase to produce an excellent control sample which lacked an active receptor. Furthermore, pre-coating the erythrocytes with an anti-GYPA monoclonal antibody, which has previously been shown to block PfEBA175 binding [8], provided an additional control to increase confidence that the measured signal was due to a specific interaction.

**Figure 3:**
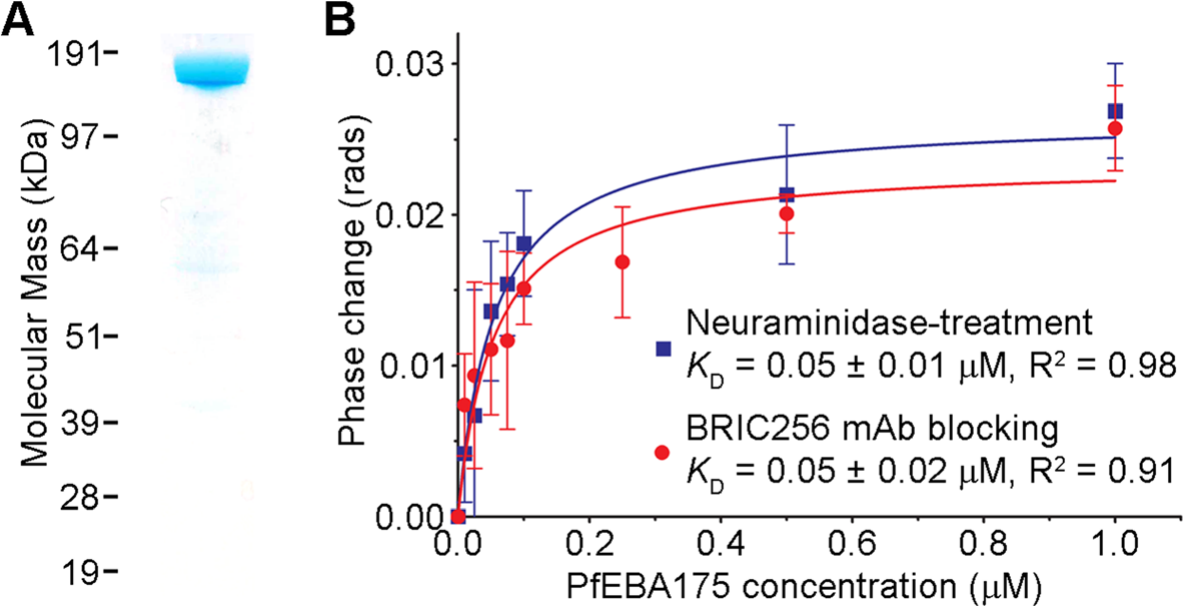
**Backscattering interferometry detects the PfEBA175**-**GYPA interaction on intact unmodified erythrocytes. (A)** The entire ectodomain of PfEBA175 was expressed and purified as a Cd4-tagged monomer and resolved under reducing conditions by SDS-PAGE. Coomassie staining revealed a single band of the expected size (185 kDa). **(B)** Quantitation of PfEBA175 binding to intact unmodified erythrocytes at equilibrium using BSI. Erythrocytes that were either neuraminidase-treated (blue squares) or pre-coated with anti-GYPA monoclonal antibody BRIC256 (red circles) were used as control samples. Data points are means ± SD from three independent experiments.

When the entire extracellular region of PfEBA175, expressed in HEK293 cells, and purified as a monomeric Cd4-tagged recombinant protein, (Figure 3A) was combined with untreated whole erythrocytes, a robust BSI phase change was observed within the ligand dilution series. To ensure that this interaction was specific and to quantify the binding signal, we used neuraminidase-treated erythrocytes as control samples. Subtraction of the query and control samples for a dilution series of the PfEBA175 protein produced a saturation binding isotherm (Figure 3B) from which a *K*_D_ of ~0.05 μM was calculated. Previously, using the same recombinant PfEBA175 protein and surface-immobilized GYPA extracted from erythrocytes and surface plasmon resonance, a *K*_D_ ~0.26 μM was reported [8]. This five-fold higher *K*_D_ is perhaps expected given that BSI is performed in free-solution with the receptor in its native environment, while the SPR value was derived from a surface-immobilized membrane protein. Other investigations have shown that both the physical environment and tethering can impact *K*_D_ measurements [26,27], further emphasizing the advantages of performing measurements on proteins within their native membrane environment.

To further demonstrate the specificity of the PfEBA175 binding, the anti-GYPA monoclonal antibody, BRIC256, that blocks binding of PfEBA175 [8] was used as an additional non-binding control sample. We pre-incubated the cells with a four-fold excess of a saturating concentration of BRIC256 prior to adding the PfEBA175. Again, once the control signal was subtracted from that of the test sample, a saturable binding response was obtained (Figure 3B), which is essentially indistinguishable from that obtained using neuraminidase as the reference. It is noteworthy that the *K*_D_s were found to be equal within the error of the measurement, as calculated using the two independent control samples in triplicate each day and on multiple subsequent days.

### Backscattering interferometry can detect and quantify PfRH5 binding to BSG on whole, unmodified erythrocytes

To further test the utility of BSI to investigate interactions at the surface of intact erythrocytes, the affinity for PfRH5 binding to its receptor BSG was measured. This interaction is of particular interest to the malaria research community because it is both essential and universally required by all tested parasite strains for erythrocyte invasion [9]. The PfRH5-BSG interaction is considerably more challenging to detect than the PfEBA175-GYPA interaction using typical erythrocyte binding assays, most likely because of the weaker interaction affinity [8,9], and because BSG is present at a much lower (~ten-fold) copy number per cell than GYPA [10].

**Figure 4:**
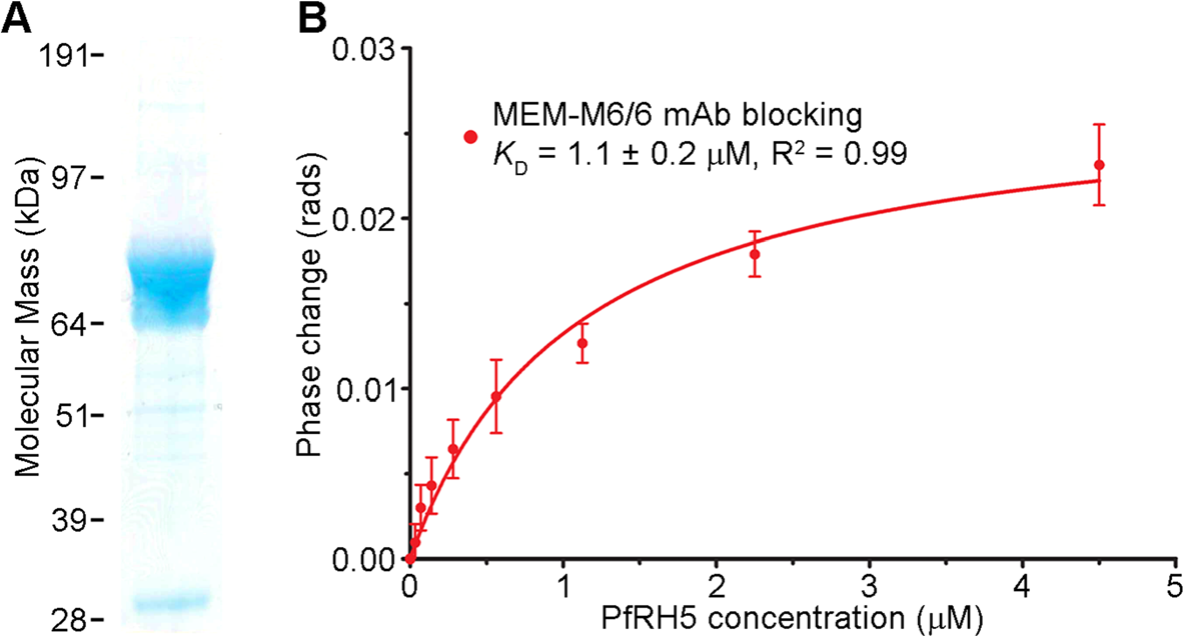
**Backscattering interferometry detects the PfRH5**-**BSG interaction on intact unmodified erythrocytes. (A)** Full-length PfRH5 was expressed and purified as a Cd4-tagged monomer and resolved under reducing conditions by SDS-PAGE. Coomassie staining revealed that the PfRH5-Cd4 preparation primarily consisted of the unprocessed full-length form. **(B)** Quantitation of PfRH5 binding to intact unmodified erythrocytes at equilibrium using BSI. Erythrocytes that were pre-coated with anti-BSG monoclonal antibody MEM-M6/6 (red circles) were used as control samples. Data points are means ± SD from seven independent experiments.

Since BSI has been shown to be exquisitely sensitive, providing femtomolar detection limits [28] and quantifying picomolar *K*_D_ values [22], equilibrium binding analyses were performed on the PfRH5-BSG interaction. Purified PfRH5 was used as the ligand (Figure 4A), and erythrocytes pre-incubated with an anti-BSG monoclonal antibody (MEM-M6/6), known to bind erythrocytes and block the binding of PfRH5 [9] was used as the control. By using a maximum concentration of PfRH5 that was four-fold in excess of the measured *K*_D_ for its receptor, a saturation binding isotherm was obtained from which a *K*_D_ of 1.1μM was calculated, a value that is identical to that previously obtained by SPR using soluble recombinant proteins [9] (Figure 4B).

## Discussion

The data presented here demonstrate that BSI is well suited for the detection and quantification of interactions between *P. falciparum* merozoite proteins and host receptors at the surface of intact, unmodified erythrocytes. BSI has numerous advantages over other approaches. First, it is easy to establish whether the interaction is saturable (and therefore specific), and does not require any washing or separation steps that may preclude the detection of transient interactions (*K*_D_ ~ μM range, t½ <1 second), a typical feature of extracellular interactions between membrane-tethered proteins [13]. Second, BSI can be used to measure interactions with unaltered cells, providing erythrocyte receptors embedded in the native membrane. This advantage is particularly important for investigating erythrocyte receptors that are very difficult to work with biochemically, particularly those that are formed from multi-chain protein complexes and/or contain multiple transmembrane-spanning regions. As demonstrated here, some erythrocyte receptors contain functionally critical post-translational modifications that are difficult to faithfully recapitulate using popular expression systems, necessitating the use of the native protein. Third, BSI is a label-free technique, so query ligands do not need to be derivatized with reporter molecules, such as fluorophores, protein tags or radioactive isotopes. These reporters lengthen assay time, increase cost and may interfere with interactions. Fourth, BSI is typically performed as a free-solution or homogeneous assay, obviating the requirement for any complex immobilization chemistry. Finally, because it is based on a microfluidic channel that has small dimensions (100 × 210μm) protein sample consumption is constrained, with required quantities in the nanomole to picomole range. Taken collectively, BSI could contribute to malaria research by both facilitating the use of recombinant, soluble *P. falciparum* merozoite protein resources [15,16] to systematically screen panels of *Plasmodium* proteins for their ability to bind erythrocytes, and performing further characterization of their receptors so as to define their mechanism of action.

One of the main motivations for exploring the use of BSI to detect interactions with receptors on the surface of erythrocytes was the difficulty in detecting binding between recombinantly expressed GYPA and PfEBA175. This is most likely due to the undersialylation of GYPA produced in HEK293E cells. Even with the additional measures taken to increase sialylation, such as co-expression with sialyltransferases, recombinant GYPA was undersialylated in comparison to the native protein, and was unable to bind to PfEBA175. Others have shown that GYPA expressed in HEK293 cells can bind to PfEBA175 [29] and so it can be speculated that the high level expression system based on EBNA1/oriP amplification used here [30] may have saturated the glycosylation pathway leading to undersialylation.

In regard to the PfEBA175-GYPA interaction, a five-fold higher equilibrium binding affinity on the surface of erythrocytes was measured using BSI relative to that measured using GYPA extracted from cells, biotinylated and immobilized on a streptavidin-coated surface for SPR experiments [8]. This higher binding affinity may be due to the increased mobility of GYPA within the erythrocyte membrane to form a PfEBA175 receptor. This observation is also consistent with both structural [31] and biochemical studies on PfEBA175 [8] which show that PfEBA175 is capable of forming dimers in solution, and that GYPA forms reversible, non-covalent dimers within the erythrocyte membrane [32,33]. These findings have led to a model whereby a GYPA dimer induces the dimerization of PfEBA175 at the merozoite surface which may be important for invasion [34]. The data presented here are consistent with this model, and suggest that the higher affinity of the PfEBA175-GYPA interaction, as measured at the erythrocyte surface rather than when tethered to a solid surface, could be due to the increased mobility of GYPA within the erythrocyte membrane, allowing it to form avid dimers.

The affinity determined here by BSI for the PfRH5-BSG interaction, with the receptor natively presented on whole erythrocytes compares very favorably to the values reported using purified recombinant soluble proteins and SPR [9,35,36]. This observation might suggest that the PfRH5 binding site on the erythrocyte surface is entirely contained within the BSG ectodomain and does not depend on other erythrocyte proteins to act as co-receptors, such as the BSG-associated monocarboxylate transporter, MCT1 [37].

## Conclusion

BSI is a method capable of detecting and quantifying molecular interactions at the surface of intact, unmodified erythrocytes. This technique has great potential as a tool to investigate the molecular basis of erythrocyte invasion, which will eventually contribute to the design of new drugs and vaccines to treat and prevent malaria.
